# Hyperpolarization of the subthalamic nucleus alleviates hyperkinetic movement disorders

**DOI:** 10.1038/s41598-020-65211-w

**Published:** 2020-05-19

**Authors:** Chun-Hwei Tai, Ming-Kai Pan, Sheng-Hong Tseng, Tien-Rei Wang, Chung-Chin Kuo

**Affiliations:** 1 Department of Neurology, National Taiwan University Hospital, Taipei, Taiwan; 2 Department of Medical Research, National Taiwan University Hospital, Taipei, Taiwan; 3 Department of Surgery, National Taiwan University Hospital, Taipei, Taiwan; 4 Institute of Physiology, College of Medicine, National Taiwan University, Taipei, Taiwan

**Keywords:** Huntington's disease, Huntington's disease, Huntington's disease, Parkinson's disease, Parkinson's disease

## Abstract

Modulation of subthalamic nucleus (STN) firing patterns with injections of depolarizing currents into the STN is an important advance for the treatment of hypokinetic movement disorders, especially Parkinson’s disease (PD). Chorea, ballism and dystonia are prototypical examples of hyperkinetic movement disorders. In our previous study, normal rats without nigro-striatal lesion were rendered hypokinetic with hyperpolarizing currents injected into the STN. Therefore, modulation of the firing pattern by injection of a hyperpolarizing current into the STN could be an effective treatment for hyperkinetic movement disorders. We investigated the effect of injecting a hyperpolarizing current into the STNs of two different types of hyperkinetic animal models and a patient with an otherwise uncontrollable hyperkinetic disorder. The two animal models included levodopa-induced hyperkinetic movement in parkinsonian rats (L-DOPA-induced dyskinesia model) and hyperkinesia induced by an intrastriatal injection of 3-nitropropionic acid (Huntington disease model), covering neurodegeneration-related as well as neurotoxin-induced derangement in the cortico-subcortical re-entrant loops. Delivering hyperpolarizing currents into the STN readily alleviated the hyperkinetic behaviors in the two animal models and in the clinical case, with an evident increase in subthalamic burst discharges in electrophysiological recordings. Application of a hyperpolarizing current into the STN via a Deep brain stimulation (DBS) electrode could be an effective general therapy for a wide spectrum of hyperkinetic movement disorders.

## Introduction

Movement disorders may be divided into two broad categories: hypokinetic and hyperkinetic. Hypokinetic movement disorders are characterized by bradykinesia and rigidity, the cardinal symptoms of Parkinson’s disease (PD). On the other hand, hyperkinetic movement disorders such as chorea and ballism constitute a more heterogeneous group of motor abnormalities characterized by uncontrollable and unwanted movements usually added to normal voluntary actions^1,2^. Pathophysiologically speaking, the cortico-subcortical re-entrant loops (i.e., the corticobasal ganglia-thalamus-cortex circuits) have been widely recognized to play a key role in the genesis of different kinds of movement disorders^1^. The cortico-subcortical re-entrant loops are classically viewed as the assembly of two major functional circuitries, the direct and indirect pathways. Motor signals generated by the motor cortex are sent into the basal ganglia and processed by re-entrant loops, in which the direct and indirect pathways presumably have facilitatory and inhibitory effects on motor activities, respectively^2,3^. The functional balance between these two pathways is thus imperative for normal signal transmission from the basal ganglia and thus normal motor presentations. An imbalance between the two pathways caused by deficient and excess dopamine, one of the most prominent neuromodulators in this regard, would then be responsible for the prototypical hypokinetic and hyperkinetic movement disorders, respectively^4,5^. A vivid clinical demonstration of the foregoing conceptual scheme can be seen in initially hypokinetic parkinsonian patients and animals who become hyperkinetic with chorea and ballism later after receiving excessive dopaminergic therapy^5,6^.

The recent success of deep brain stimulation (DBS) of the STN, a structure that is not very emphasized in the classic model, in the treatment of PD has strongly implicated a pivotal role of the STN in the functional operation of the cortico-subcortical re-entrant loops^7^. The highlighted role of the corticosubthalamic or “hyperdirect” pathway in movement disorders is further substantiated by the amelioration and induction of parkinsonian locomotor deficits via local application of NMDA receptor blockers to the STN and optogenetic excitation of the cortico-subthalamic transmission, respectively^8^. In essence, PD is a disease caused by a deficiency of dopamine (a chemical substance). The symptomatic relief of PD by injection of electrical currents (a physical maneuver) thus is itself rather intriguing. STN neurons fire in two basic modes (or patterns), namely, the tonic (or relay) mode and the burst (or oscillatory) mode. The switch between modes is chiefly determined by the membrane potential, which is in turn dependent on the ambient neuromodulators or neurotransmitters such as dopamine, glutamate and GABA^9–11^. Briefly, membrane depolarization or membrane hyperpolarization either decreases or increases the availability of T-type Ca2^+^ and Na^+^ channels (which are the key elements for the genesis of burst discharges), causing spike or burst mode discharges of the STN, respectively^10,11^. Dopaminergic input tends to result in the depolarization of STN neurons, and thus, an increase in burst discharges has been a common finding in dopamine-deprived states such as PD^12^. We have recently established a causal relation between increased burst charges of the STN and locomotor deficits in PD^10,11^. Drugs and other maneuvers (such as local application of negative currents that would depolarize the neuron) inhibiting subthalamic T-type Ca^2+^ or Na^+^ channels could therefore effectively decrease burst discharges and ameliorate parkinsonian locomotor deficits^10,11,13^. Most interestingly, local application of positive currents can hyperpolarize STN neurons, increase burst discharges and readily render a normal animal hypokinetic (i.e., create a parkinsonian state with intact dopaminergic innervation)^11^.

Despite the prominent success of DBS therapy on prototypical hypokinetic movement disorders such as PD, the treatment of hyperkinetic disorders with similar maneuvers has not been established^14–17^. Currently, the globus pallidus internus (GPi) is considered the target of choice for DBS for treating hyperkinetic movement^17^, although the STN has also been extensively explored. These studies, however, have very inconsistent results, and the application of DBS has not been generally effective. For example, Biase and Munhoz reviewed the effect of deep brain stimulation for hyperkinetic movement disorders, the average improvement from bilateral GPi DBS for tardive dyskinesia was 61%, and the average improvement of the chorea subscore after GPi DBS for Huntington’s disease was 58%^18^. However, in a previous study of GPi DBS for dystonia/choreoathetosis symptoms in cerebral palsy patients, the improvement rate varied from no improvement to a maximum of 55%^17^. In studies of STN DBS in the treatment of cervical dystonia, the effect ranged from 31.4% to 61.7%^14–18^. In this regard, one may note that the selection of these treatment targets, i.e., the GPi or STN, was mainly based on the key role of both the GPi and STN in the indirect pathway, which may have an inhibitory effect on excessive movement according to the classic model. Most importantly, a close examination of the protocols of these studies reveals that in most cases, essentially the same settings and especially the same polarity as in the stimulation for PD were used (e.g., frequency 130 Hz, pulse width 60 μs, negative polarity). Although hyperkinetic movement disorders are quite heterogeneous in both pathophysiology and clinical manifestation, it is plausible that a relative excess of dopaminergic function may play a key role in the symptomatic pathogenesis in many prototypical cases of chorea, ballism and so on, especially those showing a favorable response to dopamine antagonists. With the establishment of the molecular and cellular mechanisms underlying DBS of the STN for PD^10,11,13^ and the direct observation that the hyperpolarization of STN neurons would make the animal hypokinetic^11^, it would be highly desirable to explore the therapeutic effect of a novel STN DBS protocol with hyperpolarizing currents on hyperkinetic disorders.

## Methods

### Animals

Experiments were performed in adult male Wistar rats weighing 260–380 gm. Animals were housed at constant temperature and humidity under a 12 h light/dark cycle and free access to food and water. All animal experimental procedures were approved by the NTUCM Laboratory Animal Center. All experiments were carried out in accordance with the Animal Protection Act of Taiwan (Amended date Dec.26,2018).

### Levodopa-induced hyperkinetic model in parkinsonian rats

Rats were anesthetized with Zoletil/Xylazine (20~40 mg/kg Zoletil and 5~10 mg/kg Xylazine) (Virbac, France), mounted in a stereotactic frame (Kopf Instrument, USA) and pretreated with desipramine hydrochloride (25 mg/kg, i.p.; Sigma, USA) to protect noradrenergic neurons. After a thirty-minute interval, a hole was drilled above the right medial forebrain bundle (MFB) region, and a stainless steel cannula connected by a polyethylene catheter to a Hamilton microsyringe driven by an infusion pump (Harvard Apparatus, UK) was inserted into the right MFB (AP −2.4 mm, ML + 2.2 mm, DV −8.0 mm from bregma) according to the Paxinos and Watson stereotactic brain atlas. Five milligrams of 6-hydroxydopamine (6-OHDA;Sigma, USA) was dissolved in 0.01% ascorbic acid (Sigma, USA) and diluted with 2 ml of normal saline, providing a solution containing 10 mM 6-hydroxydopamine and 0.01% (w/v) ascorbic acid. Four microliters of 6-OHDA was infused over a period of 8 minutes. The cannula was then left in place for 10 minutes before being slowly withdrawn. After the 6-OHDA injection, the rats were kept in a cage with a warm blanket to prevent loss of body temperature until recovery (regaining of the righting reflex). Analgesics were administered immediately after surgery, and surgical wounds were observed daily for one week. The rats were tested for rotational behavior 7 to 10 days after 6-OHDA infusion by subcutaneous injection of apomorphine (0.05 mg/kg s.c.). Only rats that showed consistent turning (more than 25 turns in 5 minutes) toward the contralateral side to the 6-OHDA lesion were considered to have successful substantia nigra pars compacta (SNc) lesions (>90% dopaminergic axonal degeneration).

**Figure 1:**
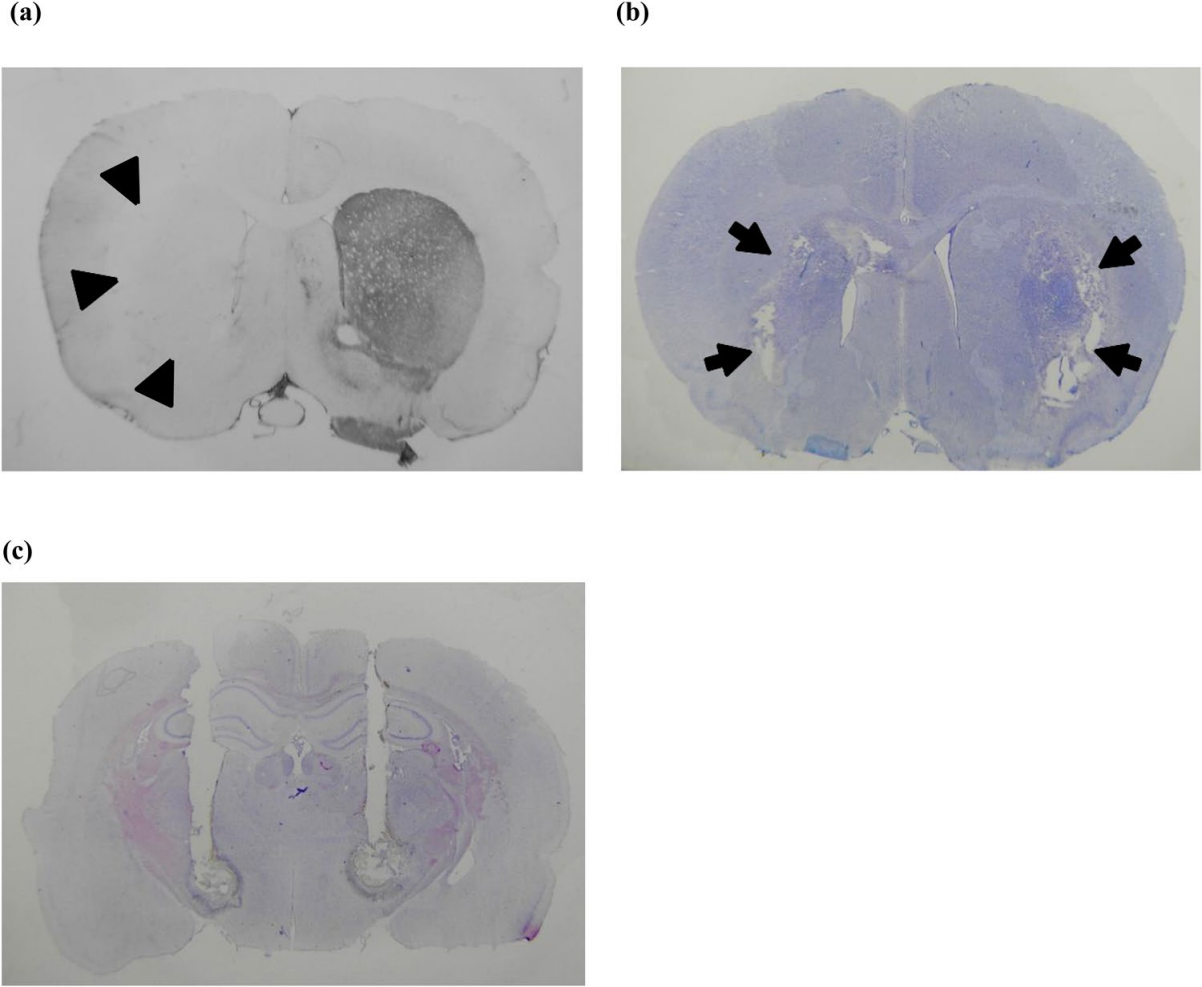
Histological verification of animal models of hyperkinetic movement. (**a**) Tyrosine hydroxylase immunohistochemistry showing unilateral deprivation of dopaminergic innervation of the striatum (arrowheads) in a parkinsonian rat with levodopa-induced dyskinesia. (**b**) Bilateral striatal lesions (arrows) in a rat with hyperkinesia after direct intrastriatal 3-NP injection shown by cresyl violet staining. (**c**) Cresyl violet staining showing trajectories of the combined microinjection cannula/stimulation electrode in the bilateral subthalamic region in a levodopa-induced dyskinesia parkinsonian rat.

All of the rats were verified for the extent of loss of dopaminergic neurons in the SNc by tyrosine hydroxylase immunohistochemistry (IHC) after completion of the experiments (Fig. 1(a)). Tissue collection for IHC was typically performed 4–6 weeks after successful 6-OHDA injection. Two weeks after the successful creation of 6-OHDA lesions, a mixture of 20 mg levodopa/kg (Sigma, USA) and 5 mg benserazide/kg (Sigma, USA), both dissolved in saline, was injected into the parkinsonian animals via subcutaneous route every 2–3 days to induce dyskinesia (i.e., 3 injections a week, for a total of 4–6 induction injections). After the third or fourth administration of the levodopa/benserazide mixture, the parkinsonian animals usually showed levodopa-induced dyskinesia, which was assessed by the abnormal involuntary movements(AIMs) score. Basically, the AIMs score comprises a rating scale for 4 subtypes of abnormal movement, namely, limb, axial, orolingual and locomotive. However, we took only the first 3 measures for a more specific evaluation of dyskinesia^19^. The behavioral and electrophysiological data in the qualified rats (those with a total AIMs score 20, 40, 60,80,100 and 120 minutes after injection of levodopa ≥ 35) were then documented with a series of challenge injections (once every 2 or 3 days for a maximum of 8–10 challenge injections)^19,20^.

### Behavior test for levodopa-induced hyperkinesia with DBS of STN

Each rat was individually placed in a transparent cage with flat lids (20 cm (w) × 36 cm (l) × 18 cm (h)) for the behavior test. During the test sessions, stimulation electrodes (approximate resistance: 10 kilohm) were connected to a 4-channel single-brush commutator (Plastics One, USA), which was then connected to a computer-controlled stimulator. A mixture of 20 mg levodopa/kg (Sigma, USA) and 5 mg benserazide/kg (Sigma, USA) was injected into the parkinsonian animals subcutaneously. Motor behaviors were assessed by the AIMs rating scale for rodents. Electrical stimulation with constant positive current was delivered into the bilateral STN during levodopa-induced dyskinesia. Each stimulation/recording session started 20, 40, 60, 80, 100 and 120 minutes after a challenge injection and lasted for 5 minutes. During each period, a blinded rater scored the rat AIMs in the first 1 minute based on the dyskinesia behavior induced by levodopa administration, followed by delivery of electrical currents into the bilateral STN for 3 minutes (the “incubation period”), and the same blinded rater scored the rat AIMs again with stimulation for another minute. After the end of the 5-minute session, the rat stayed in the observation chamber for another 15 minutes before the next stimulation/recording session started. Animal movements were continuously recorded by a digital video recorder (JVC, Japan) for subsequent offline analysis.

### 3-NP striatal neurodegeneration-induced hyperkinetic model and locomotor behavioral test

Under Zoletil/Xylazine anesthesia, 2 µl of 1 µM 3-nitropropionic acid (3-NP, 99% pure; Sigma, USA) dissolved in saline was directly injected into the bilateral striatum (AP + 0.7 mm, ±ML 2.6 mm, DV −4.5 mm from bregma) of 9–10-week-old rats at a rate of 0.5 µl/min for 4 minutes to produce excitotoxic-like lesions in the bilateral striatum. Implantation of bilateral STN chronic stimulation electrodes was performed at the same time as the surgery for creating excitotoxic lesions. The stimulation electrodes (Plastics One, Roanoke, VA) were made of polyimide-insulated stainless steel (length 10 mm; diameter 0.250 mm) and were affixed to a threaded plastic pedestal with a stainless steel ground wire (diameter 0.125 mm). The ground wire was attached to a skull screw. The electrode pedestal was then fixed onto the rat head by bone cement, which was attached to 2–3 skull screws for fixation and chronic use. One week after the formation of bilateral 3-NP lesions, the rats were tested for locomotor behavior in an open field arena. The locomotor behavior tests were performed at a fixed time (i.e., 14:00~16:00) each day for a period of 1~2 weeks. After connection of the implanted stimulation electrode to a cable, each rat was habituated for 15 minutes in a quiet plastic chamber, and then the rats were individually placed in the open field arena (45 cm (w) × 45 cm (l) × 40 cm (h)) for the locomotor behavior test. For the intrastriatal 3-NP lesion-induced hyperkinesia model, we monitored 3 parameters of locomotor activity, including total movement distance, total movement duration and rearing score during the 5-minute test period. Specifically, the rats had two sessions of locomotor behavior tests on an experimental day, the first session with sham stimulation and the second with electrical stimulation, each lasting for 8 minutes. In each session, the experimental animal was first given a 3-minute stimulation (sham or constant positive current) in an incubation chamber, followed by another 5-minute test period, when the animal was placed in the center of arena and recorded to monitor locomotor activity under the two stimulation conditions. The amplitude of the constant positive current was set at 0 (sham stimulation), 75, 150, 300 or 450 µA. The locomotor activities of the rats were recorded by a digital video recorder (JVC, Japan) and analyzed using an Ethovision system (Noldus, Netherland). All of the rats were verified for the extent of neuronal loss in the striatum after completion of the experiment (Fig. 1(b)).

### Implantation of the electrodes and electrophysiological recordings of the STN

Under Zoletil/Xylazine anesthesia, the rats were implanted bilaterally with chronic metal stimulation electrodes (PlasticOne, USA) at the stereotaxic coordinates of the rat STN (AP + 3.8 mm, ML ± 2.4 mm, DV −7.5 mm from bregma) according to the Paxinos and Watson stereotactic brain atlas. The metal stimulation electrodes were monopolar with the reference pole fixed onto a screw located ipsilaterally on the skull. The effect of electrical stimulation on hyperkinesia in these rats was examined by either AIMs score or open field activity tests at least one week after the implantation surgery. The metal electrode was connected to a computer-controlled stimulator (STG3004, Multichannel systems, Germany). For recording STN discharges during constant positive current stimulation in the hyperkinetic animal models, the skull was exposed, and the concentric metal electrode (NEX-1000) was placed into the STN with a 20° tilt in the sagittal plane under Zoletil/Xylazine anesthesia. For single-unit recordings, a bundle of 4 insulated stainless-steel electrodes (0.002 inch diameter, no. 304; California fine wire) were inserted perpendicularly into the STN. In the levodopa-induced hyperkinetic model, single-unit recordings from the STN were obtained over a 5-minute session at baseline before levodopa injection and 0, 20, 40, 60, 80, 100 and 120 minutes after levodopa injection, with or without concurrent constant positive current stimulation. In the 3-NP-induced hyperkinetic model, each recording session was performed at baseline (i.e., before stimulation for 5 minutes), during constant current stimulation (5 minutes) and post-stimulation (four 5-minute sessions, measurement done in the 4th session). The constant current stimulation did not produce stimulation artifacts during the single-unit recordings. At the end of the recording session, the rats were deeply anesthetized and perfused intracardially with 10% formalin. The brain of each of the rats was removed for histological verification and electrode localization (Fig. 1(c)). The electrophysiological data were postprocessed with the spike detection and sorting tools of SciWorks 5.0, USA. A spike was detected whenever a negative deflection of the potential crossed the predefined voltage threshold. The detected spikes were further sorted with principal component analysis (PCA) as stated in our previous study^10^. The selected single units were then categorized ashaving tonic or bursting activity according to their firing pattern. For example, burst activity in a single unit was defined by interspike intervals of less than 20 milliseconds for at least four consecutive spikes^10^.

### Histology and immunohistochemistry

Free-floating serial sections of the substantia nigra and the striatum were processed for tyrosine hydroxylase immunohistochemistry. Briefly, brain sections were first preincubated with 3% normal horse serum in Tris buffer solution (TBS, Sigma-Aldrich) containing 0.3% Triton X-100 (TBST, Sigma-Aldrich) for 30 minutes at room temperature, and then incubated in TBST containing 1% normal horseserum for 30 minutes and finally in TBST containing monoclonal antibody to tyrosinehydroxylase (1:500, RRID: AB_477560, Sigma-Aldrich) for 24 hours at 4 °C. After washing in TBST, the brain sections were incubated with biotinylated anti-mouse IgG(1:500, RRID: AB_258619, Sigma-Aldrich) for 2 hours at room temperature, rinsed again in TBSTcontaining 1% normal horse serum for 1 minute and then incubated with ExtrAvidinperoxidase (1:500, Sigma-Aldrich) for 1 hour at room temperature. These sections were there after exposed to a mixture of 3, 3-diaminobenzidine, H_2_O_2_, and Ni^2+^ (DBA kits; VectorLaboratories), fixed on gelatin-coated slides, dehydrated with alcohol in ascending concentrations, cleaned in xylene, mounted with Permount (Fisher Scientific), and covered with cover glass for histochemical examination.

### Human subject

The 62-year-old male PD patient underwent standard procedures of pre-DBS evaluation and DBS implantation surgery after informed consent from the patient and his family under the regulation of Ministry of Health and Welfare of Taiwan. At post-operative period, due to the falling accident and emergent condition, another informed consent (both oral and written) regarding to the special DBS programming procedure in this study were obtained from the patient and his family. Ethical approval of administration office was not sought in this case because according to the Regulation of Research Ethics Committees of National Taiwan University, report of one to three clinical experiences or cases identified in the course of clinical care do not require special approval. In addition, informed consent relating to publication of identifying informations and images/videos of this patient in an online open-access publication had also been obtained from the patient and his family.

### Statistics

*In vivo* numerical data and statistical analyses were managed with Prism software (GraphPad Software). The behavior data shown in Fig. 5(c–e) were analyzed using unpaired (2-tailed) Student’s *t* tests. The data in Figs. 3(c), 4(d–f), 6(c–e), and 7(d–g) were compared with 1-way ANOVA followed by Dunnett’s test. All data are presented as the mean ± SEM. For all comparisons, a *P* value less than 0.05 was accepted as indicative of significant differences.

## Results

Our results showed injecting hyperpolarizing current into the STN of two different hyperkinetic animal models, namely, L-DOPA-induced dyskinesia model and Huntington disease model, caused significant alleviation of animal hyperkinetic movement disorder symptoms, and also changes in electrophysiological findings. And these findings could also be applied in one human  patient with hyperkinetic movement disorder.

### Amelioration of levodopa-induced hyperkinesia by delivery of positive currents into the STN

**Figure 2:**
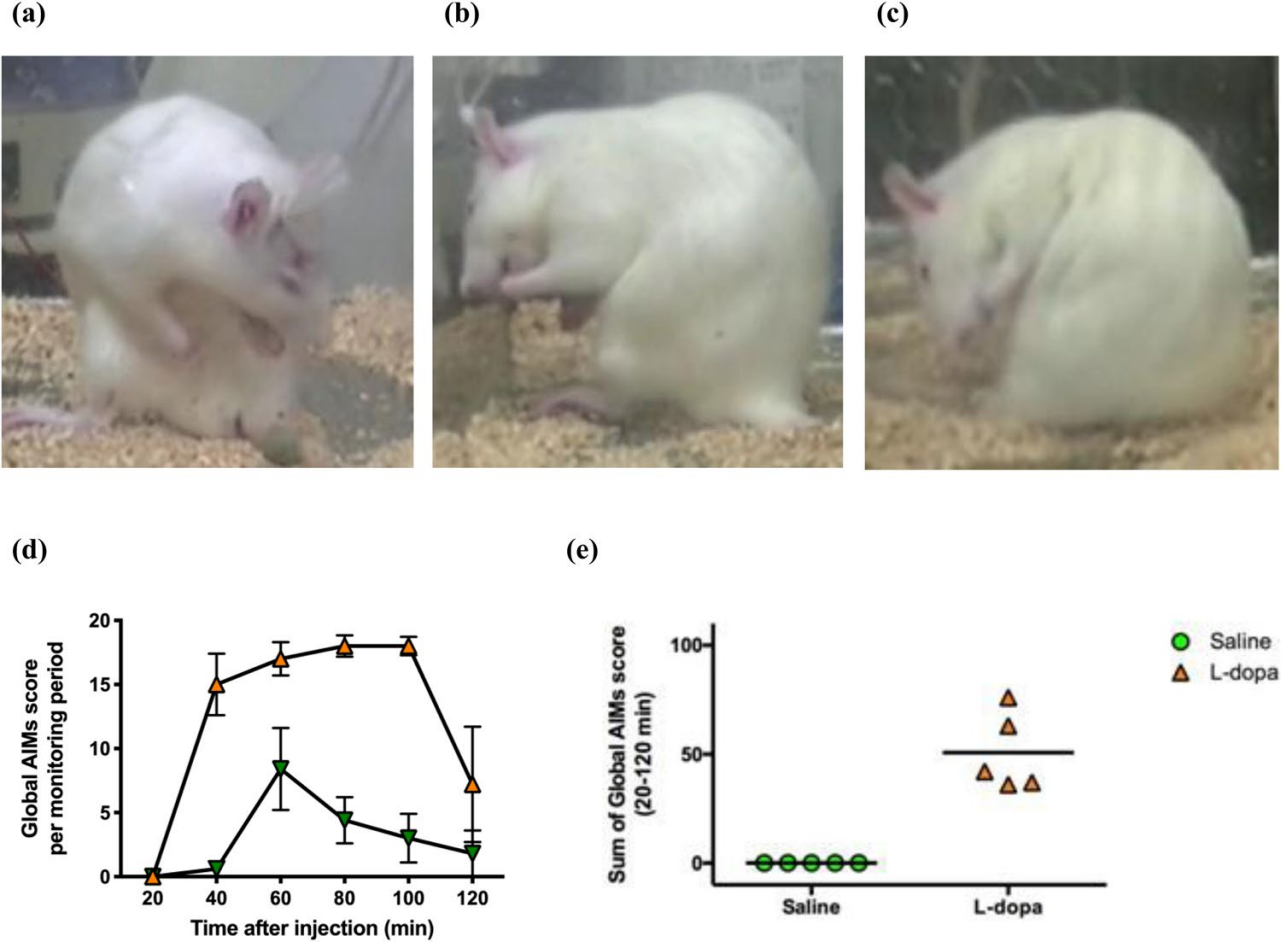
L-dopa-induced dyskinesia in parkinsonian rats. (**a**–**c**) Examples of levodopa-induced dyskinetic movement in parkinsonian rats after repeated injection of L-dopa every other day for 6–8 consecutive days. Video recording for calculating global abnormal involuntary movements (AIMs) scores demonstrating the following AIMs scoring items: (**a**) axial dyskinesia (tortuous truncal movement), (**b**) limb dyskinesia (repeated forelimb movement) and (**c**) orofacial dyskinesia (repeated jaw opening and tongue protruding). (**d**) Time courses of the global AIMs score (mean ± SEM) at 20, 40, 60, 80 and 120 minutes, successfully showing the induction of dyskinesia by L-dopa in five parkinsonian rats. (**e**) The sum of the global AIMs scores in the same rats after induction of dyskinesia either by L-dopa or saline.

**Figure 3:**
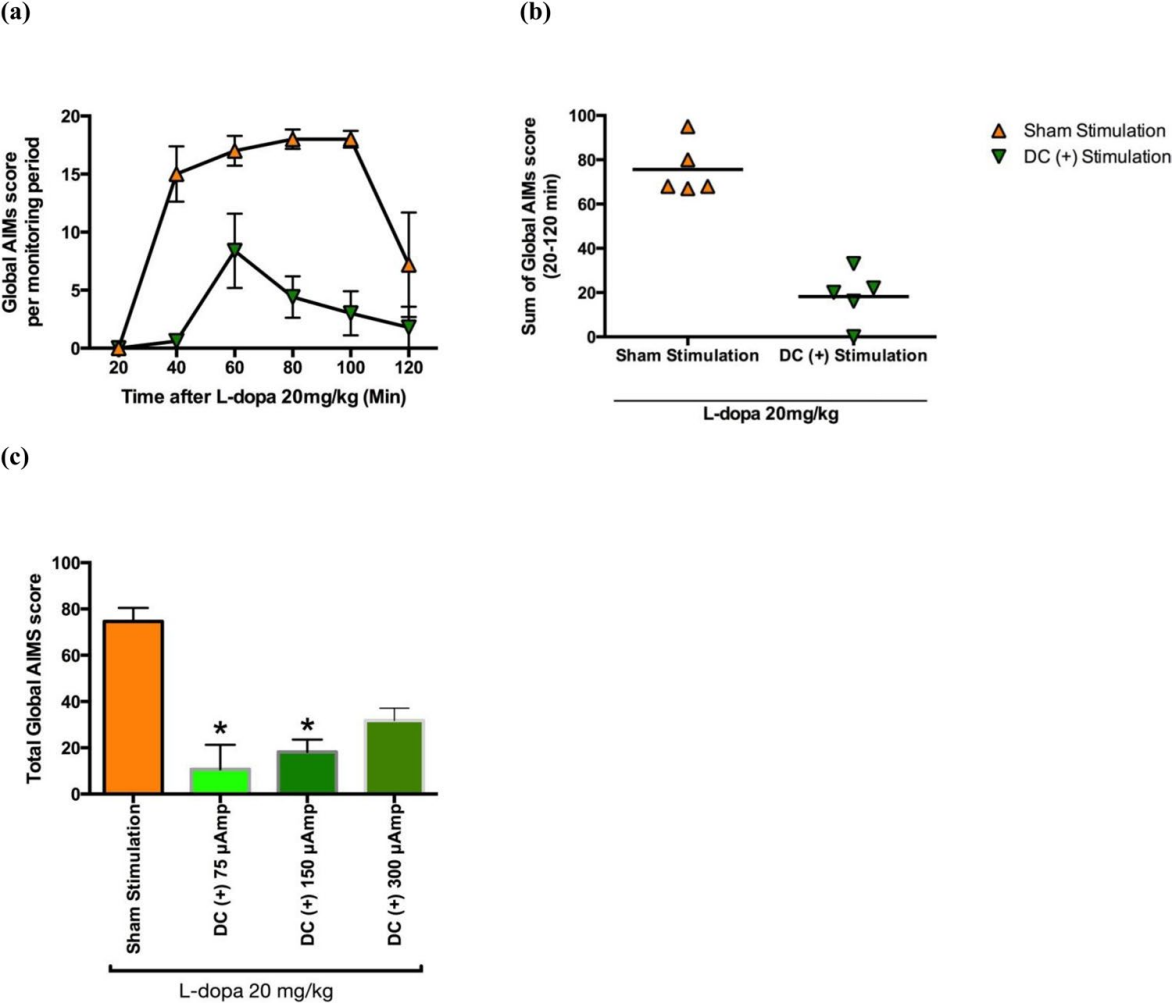
The effect of delivery of constant positive current into the bilateral STN on L-dopa-induced dyskinesia in parkinsonian rats. (**a**) Time courses of AIMs score (mean ± SEM) at 20, 40, 60, 80 and 120 minutes after two consecutive L-dopa challenge injections, one with and the other without delivery of constant positive current (150 µA) into the bilateral STN in the same rats as those in Fig. 2. (**b**) The sum of the global AIMs scores in these parkinsonian rats at baseline and during bilateral STN injection of constant positive current (150 µA). (**c**) Changes in the sum of the global AIMs scores in L-dopa-induced dyskinesia parkinsonian rats during sham and bilateral STN constant positive current injection at 75, 150 and 300 μA (n = 5, *p < 0.05).

**Figure 4:**
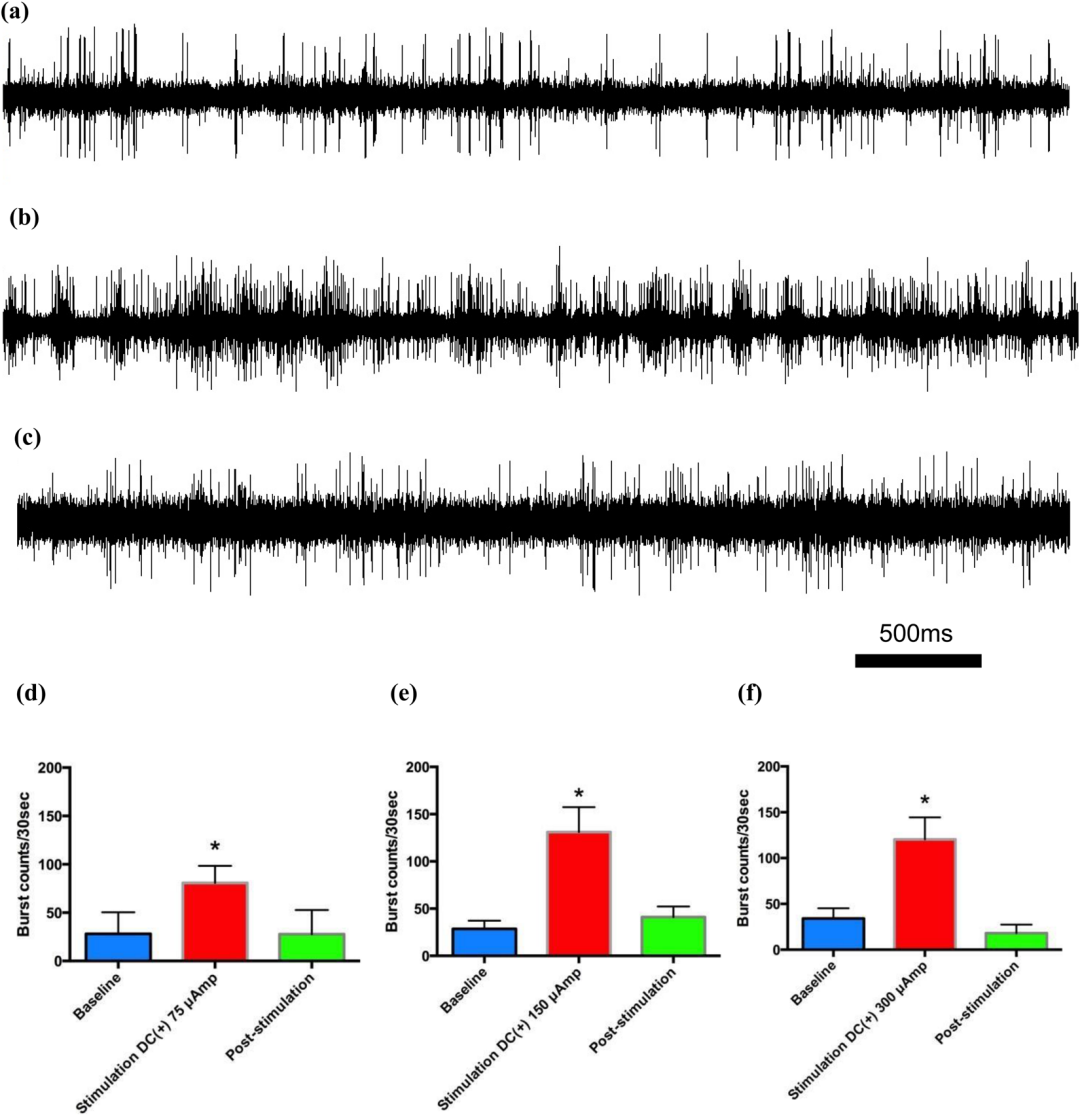
The effect of constant positive current delivered into the STN on the electrophysiological activity of subthalamic neurons *in vivo* in parkinsonian rats with L-dopa-induced dyskinesia. Sample sweeps of the STN firing pattern (**a**)before, (**b**)during and (**c**) 5 minutes after cessation of 150 µA of constant positive polarity current into the STN. The number of burst firing counts per 30 seconds had already increased significantly with (**d**–**f**) 75 µA of current (n = 6) and increased even more with 150 µA (n= 7) or 300 µA (n = 3) of constant current delivered into the bilateral STN (*p < 0.05).

In parkinsonian rats, we established a levodopa-induced hyperkinetic model involving orofacial/limb/axial dyskinesia (n = 6, Fig. 2(a–c)), which can be shown by the marked elevation of the sum of the global AIMs score between 20~120 minutes after a challenge levodopa injection compared to saline injections (n = 6, Fig. 2(d,e)). After the establishment of the hyperkinetic movement disorder, constant positive currents of different amplitudes were injected into the bilateral STN. The global AIMs scores were correlatively and markedly reduced by direct injection of constant positive currents into the bilateral STN in comparison with the sham stimulation group (n = 5, Fig. 3(a,b)). Positive currents of 75 µA(n = 6), 150 µA(n = 7), and 300 µA(n = 3) showed a rough but not statistically significant “dose-dependent” trend of the effect on AIMs score. Delivery of constant positive currents readily increased subthalamic burst discharges, an effect that diminished quickly (within 5 minutes) after turning off the currents (Fig. 4(a–f)). Although there was a definite behavioral as well as electrophysiological effect of electrical stimulation, there was no clear-cut dose dependence for the injected currents. This may be related to the fact that the burst discharges were increased by increasing hyperpolarization only within a certain window, and excessive hyperpolarization decreased rather than increased the autonomous discharging of the STN (see Discussion).

### Amelioration of hyperkinesia induced by bilateral intrastriatal injections of 3-NP by delivery of a positive current into the STN

**Figure 5:**
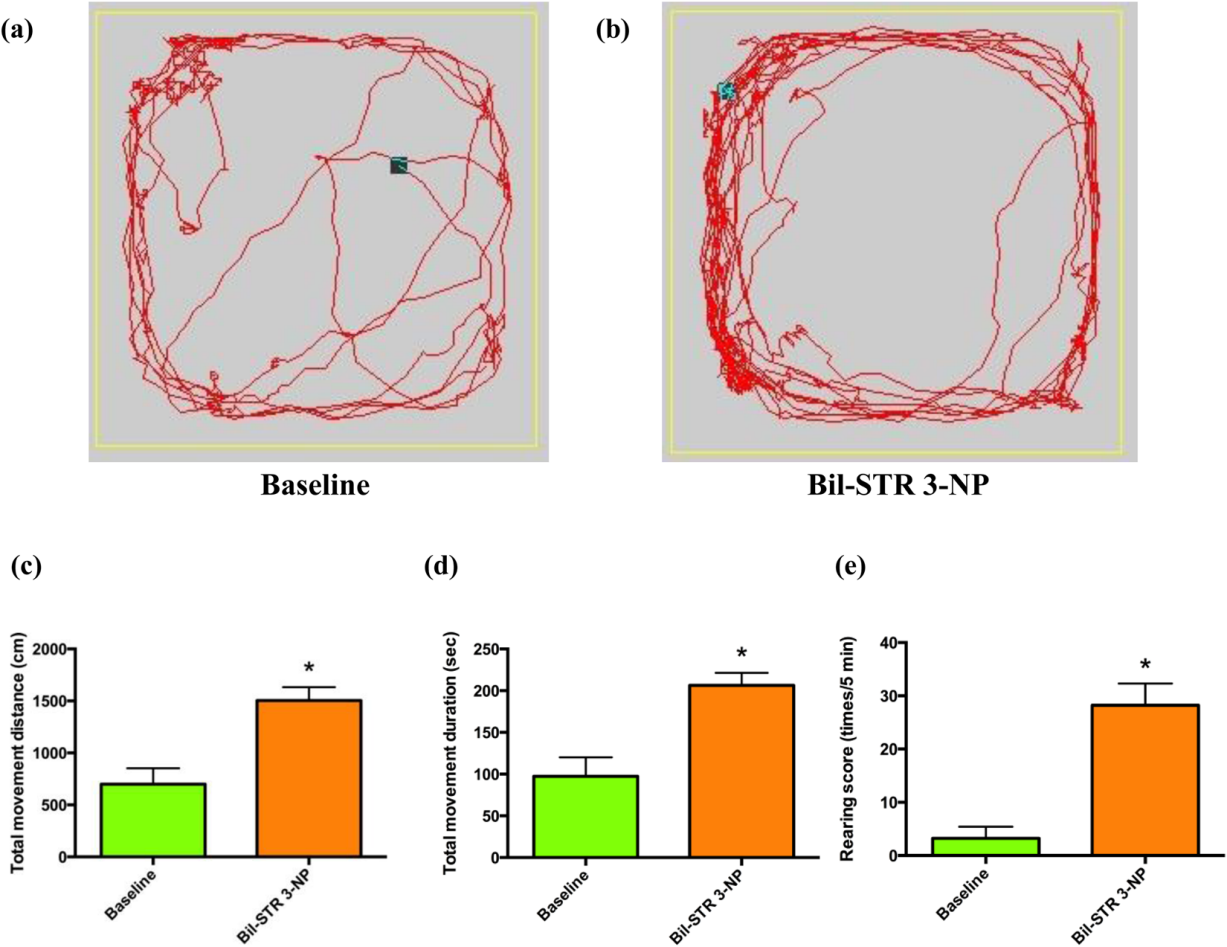
The hyperkinetic animal model induced by injection of 3-NP into the bilateral striatum. The sample traces of locomotor activity in a rat (**a**) at baseline and (**b**) one week after 3-NP injection. (**c**) The total movement distance, (**d**) the total movement duration, and (**e**) the rearing score are all increased one week after the injection (n = 5, *p < 0.05 compared to control).

**Figure 6:**
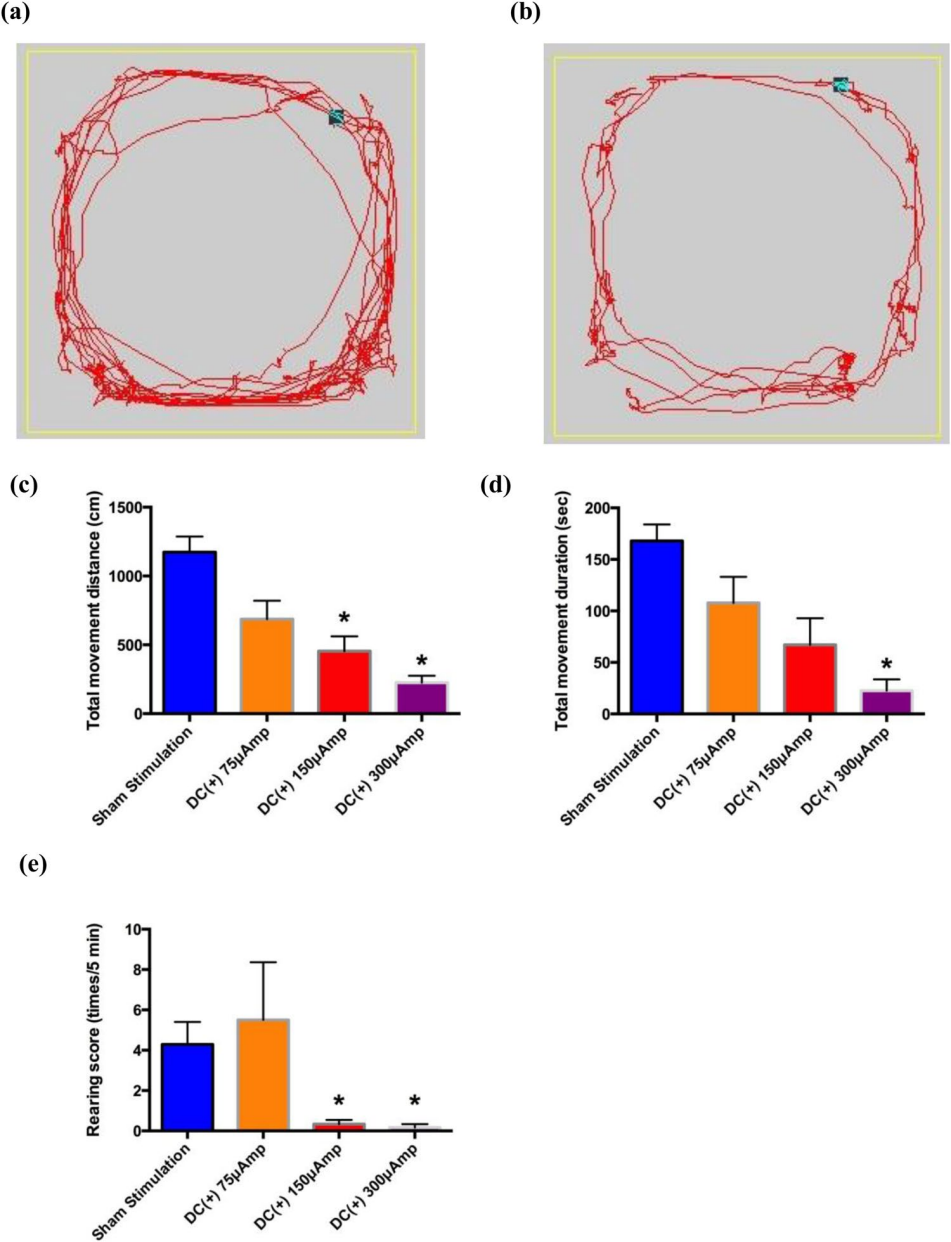
The effect of constant positive current delivered into the bilateral STN on hyperkinesia induced by bilateral intrastriatal 3-NP injection. Sample traces of locomotor activity during (**a**) sham stimulation and (**b**) delivery of 150 µA of constant positive constants into the STN. Average locomotor activity, including (**c**) movement distance, (**d**) movement duration and (**e**) rearing score, were all decreased by the constant positive current delivered into the STN (n = 18 for sham stimulation in the six rats, n = 6 for the others, *p < 0.05 compared to sham stimulation). The differences between any two of the three “doses” of the injected current, however, are not statistically significant.

**Figure 7:**
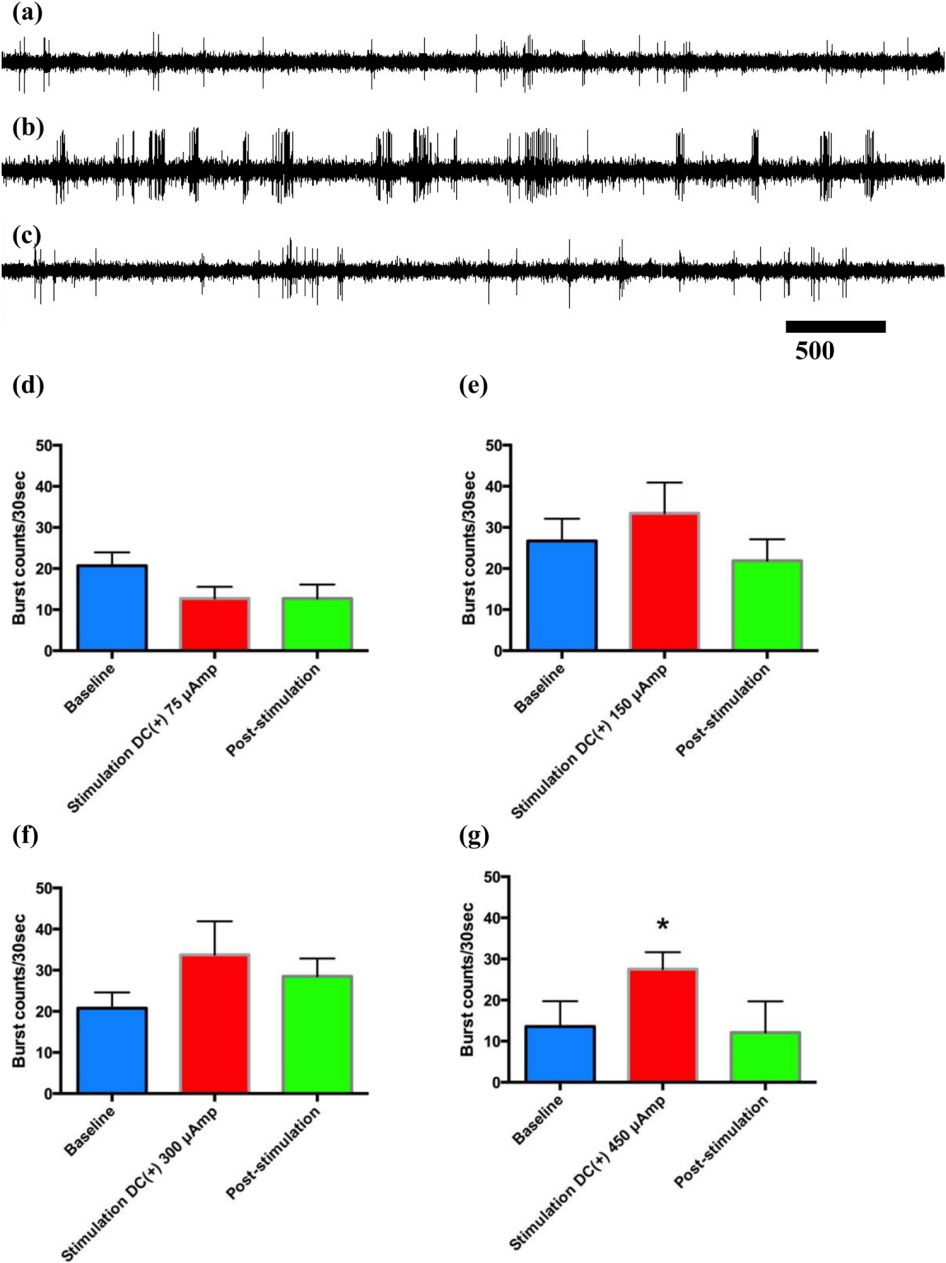
Electrophysiological changes before, during and after delivery of constant positive current into the bilateral STN in rats with 3-NP-induced hyperkinesia. Sample sweeps of the STN firing pattern (**a**) before, (**b**) during, and (**c**) after delivery of 450 µA of constant positive current into the STN. (**d**–**g**) Burst counts per 30 seconds tended to increase during stimulation with 150–450 µA current but not with 75 µA current. However, statistical significance was achieved only with 450 µA stimulation (n = 5 in two rats for each current intensity, *p < 0.05 compared with the baseline data before stimulation).

A marked increase in locomotor behavior was usually noted 3–5 days after the injection of 3-NP into the bilateral striatum, and hyperkinesia was quantitatively demonstrated by the open field locomotor activity test. There was a significant increase in the total movement distance, the total movement duration and the rearing frequency (n = 5, Fig. 5(a–d)). The increased locomotor activity was readily and evidently suppressed by injection of constant positive current into the bilateral STN (n = 18, Fig. 6(a,b)). In this case, the ameliorating effect on all of the hyperkinetic parameters was roughly correlated with amplitudes of injected currents between 75 µA (n = 5) and 300 µA (n = 5), although the “dose-dependence” was not statistically significant (Fig. 6(c~e)). The ameliorating effect of the constant positive current on hyperkinetic locomotor behaviors in the different models remained the same as long as the stimulation continued. No decay in efficacy was observed with 6–8repetitions or lengthening of the maneuver up to 15 minutes. We also performed *in vivo* single-unit extracellular recordings of the STN one week after bilateral intrastriatal injection of 3-NP in these rats. After delivery of 75 µA ~450 µA of constant positive current directly into the STN, the relationship between current and subthalamic burst count could still only be considered a trend rather than a clear-cut “dose-dependence” during the stimulation (Fig. 7(a–g)). The STN burst discharges increased immediately during constant positive current stimulation and returned to baseline shortly (within 5 minutes) after cessation of constant current delivery into the STN.

### Application of positive current stimulation in a patient with hyperkinetic movements

**Figure 8:**
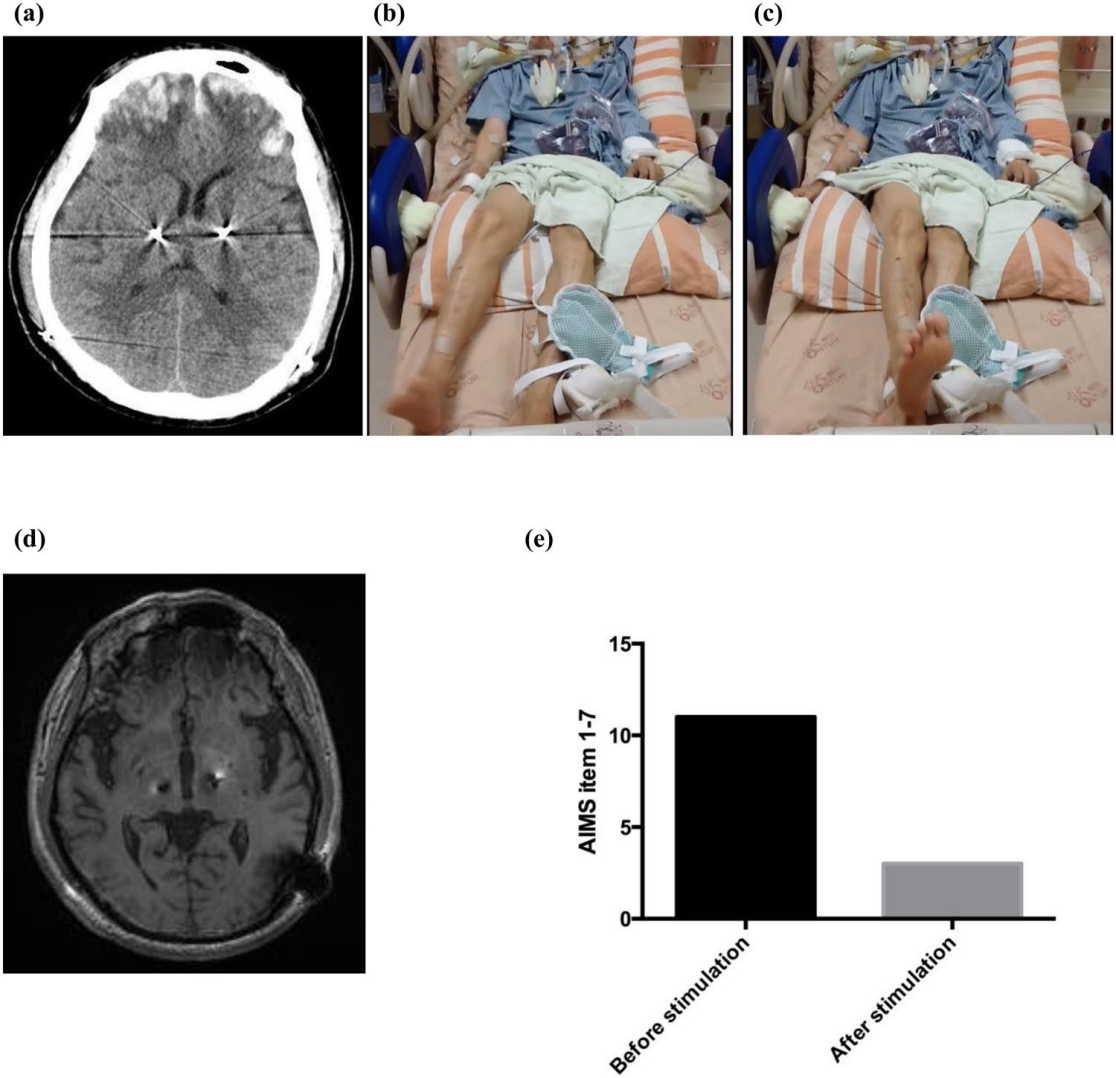
Marked amelioration of hyperkinetic movement in a patient with traumatic intracranial hemorrhage in the bilateral frontal regions by injection of positive current into the STN. (**a**) CT scan shows bilateral frontal ICH after an accidental fall with severe head injury in the PD patient just implanted with bilateral STN electrodes. (**b**) and (**c**) Photos of hemiballistic movement of the right upper and lower limbs of the patient. (**d**) Location of the DBS electrodes. (**e**) AIM scores of the patient before, during and after DBS with constant positive current (left STN stimulation with the following settings: 0(−) 1(+)2(+)3(+) 7.5 volts, 450 µs, 130 Hz). The hyperkinetic movements were always markedly inhibited “immediately” upon switching on the DBS but would recur within a few seconds after turning off the DBS. Please refer to the video segment in the supplemental material.

The patient was a 62-year-old male with advanced PD for 18 years. He had suffered from severe motor fluctuation with moderate on-time dyskinesia for 4–5 years before surgery for DBS electrode implantation. The implantation surgery itself was uneventful, but he had an accidental fall that caused severe traumatic head injury two weeks after DBS implantation surgery. A CT scan revealed bilateral frontal intracranial hemorrhage, subarachnoid hemorrhage, and skull fracture (Fig. 8(a)). Clinically, the patient presented with symptoms and signs of altered consciousness, dysphagia and increased intracranial pressure. Right hemiballism-hemichorea developed 5–7 days after the traumatic injury. The hyperkinetic disorder became increasingly severe day by day, resulting in the patient being unable to rest, intake substances orally or even breathe smoothly. Discontinuation of dopaminergic medications and daily administration of up to 15~20 mg of haloperidol to control the severe hyperkinetic symptoms was ineffective (Fig. 8(b,c)). The ballistic movement was so aggravated that not only respiration but also chest hygiene were jeopardized, causing pneumonia. He was sent to the ICU where endotracheal intubation, ventilatory support and finally generalized anesthesia were applied. With the previously described potential physiological and pathophysiological roles of the STN in mind, we reprogrammed the DBS electrode in the left STN region (Fig. 8(d)) after titration of voltage and pulse width. The hyperkinetic movement was suppressed immediately after turning on positive current injection (Fig. 8(e); supplemental video provided). In the following few days, there were only occasional breakthroughs of hemichorea-hemiballism. Most of his hyperkinetic symptoms were inhibited by reprogramming of left STN DBS between 7.5 and 9.0 volts with unchanged contacts and stimulation frequency. The hyperkinetic movement gradually disappeared by itself 10~12 weeks after onset, and the intensity of the positive polarity stimulation to the of left STN was decreased accordingly. No more hemichorea-hemiballism was observed 6 months after onset even without left STN DBS.

## Discussion

We chose a levodopa-induced hyperkinetic model in parkinsonian rats and a hyperkinetic model induced by an intrastriatal injection of 3-nitropropionic acid, endeavoring to cover a wide scope of pathological conditions related to clinical hyperkinetic disorders. Levodopa-induced dyskinesia, a the most common motor complication associated with pharmacotherapy of PD, is probably the most common among the hyperkinetic movement disorders^19,20^. The development of dyskinesia in this model is related to the deficiency of dopamine and maladaptive plastic changes in subjects receiving exogenous pulsatile levodopa supplementation. This rat model clearly demonstrates orofacial, limb and axial dyskinetic movement at least partly mimicking clinical disorders characterized by hyperkinesia or dyskinesia directly involving functional dopaminergic excess^20^. In the second hyperkinetic model, an intrastriatal injection of 3-nitropropionic acid, an environmental mitochondrial inhibitor, produces excitotoxic-like lesions of the striatum. The acute damage caused by the direct injection of 3-NP into the bilateral striatum can produce hyperkinetic movement, bilateral dystonia, general incoordination, or even cognitive deficits in animals^21,22^. The appearance of abnormally increased motor activity can be related to the hyperkinetic movement disorder observed in Huntington’s disease (HD) patients^21^, and so this model is therefore also appropriate for the examination of the effect of DBS of the STN on hyperkinetic movement. We further propose a report on a PD patient who suffered from traumatic intracranial hemorrhage and developed severe hemichorea-hemiballism. All of the foregoing hyperkinesias were readily remedied by STN DBS with positive current. These findings provide imperative novel insight into not only the clinical management of hyperkinetic movement disorders but also the basic rationales of motor control through cortico-subcortical re-entrant loops.

There are apparent differences in the pathogenesis and pathophysiology underlying the hyperkinetic movement among the two animal models and the clinical case. In the levodopa-induced hyperkinetic movement model in the parkinsonian rats, dyskinesia is triggered by pulsatile exogenous dopamine administration. There could be a relatively large-scale dopaminergic dysfunction, especially in the striatum and STN, where hypersensitivity for dopamine most likely develops after deprivation of the normally abundant input^19,23^. The altered dopaminergic effect presumably would result in decreased firings of downstream structures such as the Gpi/SNR, followed by apparently increased as well as “unchecked” thalamocortical activity^13^. These increases in unchecked “noise” in cortical motor commands could be directly responsible for the increased involuntary or hyperkinetic movements. Conventionally, DBS of the bilateral GPi has been applied to PD patients in this situation with limited or inconsistent success, presumably via modulation of the abnormal signal output from the GPi and thus a decrease in levodopa-induced dyskinesia^24,25^. The evident normalization effect of STN DBS on levodopa-induced hyperkinesia in parkinsonian rats would also suggest that appropriate modulation of STN firing itself may effectively offset the deranged striatal inhibitory drives on the GPi. The critical role of the STN in hyperkinetic disorders with a focal but distant pathogenesis in humans could be underscored by the marked improvement from the same STN DBS applied to the patient suffering from acute traumatic frontal hemorrhage-associated hemichorea-hemiballism, once more demonstrating the unparalleled ability of the STN to trim the noises found in frontal motor commands. In this regard, it is of note that 3-NP is a mitochondrial toxin that causes mitochondrial dysfunction and the preferential degeneration of medium-sized spiny GABAergic neurons in the striatum, at least in the early stage^21^. Hyperkinetic movement with increased locomotor activity is observed in the early stage after 3-NP injection, but hypokinetic movement or even akinesia commonly follows, chiefly determined by the extent and duration of striatal neuronal loss^21,22^. This course is similar to that of HD, although the exact pathogenesis of the peculiar course remains unsettled^21^. However, the evident normalization effect of STN DBS on the 3-NP model should lend strong support to the idea that modulation of STN firing itself may correct the deranged striatal drives directly originating from the degenerated striatal network.

The discharge patterns of thalamic and subthalamic neurons can be grossly divided into burst and tonic modes^11,12,26^. The former is more autonomous (i.e., less responsive to phasic input and consequently delivering more “self-driven” anomalous output to the relevant networks) and necessitates a sufficient number of hyperpolarization-activated cationic, T-type Ca^2+^, and Na^+^ channels to appear. The latter, on the other hand, relies more on extrinsic phasic inputs than intrinsic cationic currents and thus is less autonomous or more faithfully responsive to external drives^9,12^. We have demonstrated that the local application of inhibitors of T-type Ca^2+^ or Na^+^ channels decreases subthalamic burst discharges, which has a direct causal relation with the hypokinetic locomotor deficits in PD^12,13^. Moreover, depolarization of STN neurons by injection of an appropriate amount of negative current into the STN, irrespective of the frequency of the current pulses, may inactivate (or decrease the availability of) T-type Ca^2+^ and/or Na^+^channels^12^. As deprivation of the dopaminergic input in PD tends to shift the discharge pattern of the STN to burst mode^11,12^, it is plausible that negative-current DBS and the consequent neuronal depolarization may increase the inactivation of T-type Ca^2+^ and/or Na^+^ channels and thus antagonize the burst-prone discharge pattern of the STN. The STN neurons therefore would more faithfully follow the extrinsic phasic input, most importantly via the hyperdirect pathway from the frontal motor cortices. In this regard, the injection of positive currents into the STN very likely would hyperpolarize the neurons and thus increase the availability of T-type Ca^2+^ and/or Na^+^ channels because of the decreased inactivation^12,13^. STN neurons would then fire more in the burst mode (i.e., more autonomously) and less precisely respond to the phasic input from the frontal motor cortices. A decrease in locomotor behavior ensues, either remedying any pre-existing hyperkinetic disorders or giving a normal subject hypokinetic or parkinsonian characteristics^10^. The molecular mechanisms also partly explain why a strict “dose-dependence” of the effect of the hyperpolarizing currents may not have always been observed in the experiments, because the excess hyperpolarization of the STN neuron may surpass the membrane voltage range where the revived channels could cooperate with the other depolarization elements of the cell membrane to depolarize the cell to fire a burst again. In other words, an increase in the level of hyperpolarization may either increase or decrease the autonomous burst discharges. Deliberate titration of the amplitude of the positive currents therefore must be exercised for the maximal therapeutic effect on hyperkinetic movement disorders in a clinical setting.

We saw a prominent remedying effect of STN DBS with positive current on hyperkinetic movement disorders, regardless of whether they arise from a local or distant, a focal or relatively large-scale, or a neurochemical or structural derangement. Together with the well-established therapeutic effect of STN DBS with negative current on hypokinetic movement disorders such as PD, it is highly likely that the STN plays an essential and determinant “switching on/off” role in motor execution. In this regard, the reciprocal connections between the glutamatergic STN and the GABAergic GPe could be viewed as an essential complex that maintains stable baseline discharges and thus a baseline level of inhibition of thalamocortical activity via direct STN output to the GPi/SNr, which is characterized by nearly continuous high-frequency baseline discharges. The formation of a mature motor command requires the repeated circling of information in the cortico-subcortical re-entrant loops, chiefly involving the “direct” pathway from the striatum to the GPi/SNr for sharpening of the main signal and deletion of the associated noises. This formation process may take a few hundred milliseconds to complete and is manifest at the cortical level as “readiness potentials”, during which immature cortical arousals would not result in vivid motor behaviors mostly because of the strong inhibition of thalamocortical activity implemented by the hyperdirect pathway or corticosubthalamic tract^9,13^. The motor command gradually matures at the corticostriatal level with a gradual decrease in STN bursting (because of less noise from the cortex) and GPi/SNr activity (also because of the stronger and more focused inhibition from the striatum), decreasing to a point that the command can no longer be inhibited and is thus “mature” and subsequently executed. The focused and sufficiently strong striatal output may then increase STN burst activity again via the GPe and the indirect pathway, bringing back the baseline discharge patterns of the STN. This motor command is therefore over, and the system is ready for the next command. The appropriate changes in STN activity, chiefly following the drives from the hyperdirect pathway and then the indirect pathway, are therefore most important for the proper execution of motor commands. A decrease and increase in the autonomous or semiautonomous burst discharges in the STN would then be associated with a decrease and increase in locomotor activity, respectively.

The successful use of STN DBS in the treatment of both hypokinetic and hyperkinetic movement disorders, involving the previously described cortico-subcortical re-entrant loops, relies on the application of not only the appropriate magnitude but also the appropriate polarity of the current. It would be very interesting to examine and modify these novel rationales relaying cellular discharges to behaviors in additional animal models, to patients with different kinds of movement disorders, or even to patients with nonmotor impulse control or execution disorders to further elucidate the full function of the STN and the cortico-subcortical re-entrant loops. In conclusion, application of a hyperpolarizing current into the STN via DBS electrodes could be an effective therapy for a wide spectrum of hyperkinetic movement disorders.

## Supplementary information

Supplemental video segments.

Video 1.

Video 2.

Video 3.

Video 4.

## Data Availability

The datasets generated and/or analysed during the current study are available from the corresponding author on request.
