# Author Correction: Hyperpolarization of the subthalamic nucleus alleviates hyperkinetic movement disorders

**DOI:** 10.1038/s41598-020-67067-6

**Published:** 2020-06-12

**Authors:** Chun-Hwei Tai, Ming-Kai Pan, Sheng-Hong Tseng, Tien-Rei Wang, Chung-Chin Kuo

**Affiliations:** 1 Department of Neurology, National Taiwan University Hospital, Taipei, Taiwan; 2 Department of Medical Research, National Taiwan University Hospital, Taipei, Taiwan; 3 Department of Surgery, National Taiwan University Hospital, Taipei, Taiwan; 4 Institute of Physiology, College of Medicine, National Taiwan University, Taipei, Taiwan

Correction to: *Scientific Reports* 10.1038/s41598-020-65211-w, published online 19 May 2020

The Acknowledgements section in this Article is incomplete.

“CHT is funded in part by the Ministry of Science and Technology, Taiwan (MOST 104–2314-B-002–047-MY3).”

should read:

“CHT is funded in part by the Ministry of Science and Technology, Taiwan (MOST 104–2314-B-002–047-MY3. CCK is funded by the Ministry of Science and Technology, Taiwan (MOST 106-2321-B-002 -032 and MOST 107-2321-B-002-012).”

