# Supplementary material for: Hyperpolarization of the subthalamic nucleus alleviates hyperkinetic movement disorders
Source: Sci Rep. 2020 May 19;10:8278. doi: 10.1038/s41598-020-65211-w (PMC7237462; doi:10.1038/s41598-020-65211-w)
Supplement: Supplementary file 1 — Supplemental video segments. [file 41598_2020_65211_MOESM1_ESM.pdf]

Hyperpolarization of the subthalamic nucleus alleviates hyperkinetic movement disorders

Chun-Hwei Tai[1], Ming-Kai Pan[2], Sheng-Hong Tseng[3], Tien-Rei Wang[1], Chung-Chin Kuo[1,4]

[1]Department of Neurology, National Taiwan University Hospital, Taipei, Taiwan
[2]Department of Medical Research, National Taiwan University Hospital, Taipei, Taiwan
[3]Department of Surgery, National Taiwan University Hospital, Taipei, Taiwan
[4]Institute of Physiology, College of Medicine, National Taiwan University, Taipei, Taiwan

Supplemental video segments

Video Legends

Video 1. The hyperkinetic movement (hemiballism-hemichorea on the right side) of the patient was mostly resolved immediately after switching on the DBS with high-frequency, long-pulse, positive polarity stimulation (i.e., 0(-) 1(+)2(+)3(+), 7.5volts, 450μs, 130Hz) of the left STN. Mild residual chorea movement was observed in the right lower limb. On the other hand, he was able to perform a voluntary grasping movement of the right hand, which had never been observed before DBS, after the hemiballism-hemichorea was greatly alleviated by the hyperpolarizing DBS of the STN.

Video 2. Twenty-four hours after stimulation of the left STN with positive current. Although mild residual hyperkinetic movement of the right limbs could be observed, the inhibitory effect of DBS on the hemiballism-hemichorea largely persisted, enabling the patient to adequately rest his right limbs.

Video 3. At roughly the same time as video 2, the patient would always suffer from severe right hemiballism-hemichorea again if DBS of the left STN was turned off.

Video 4. One week after DBS of the left STN, the inhibitory effect on hyperkinetic movement persisted well.
